# Initial description of changes in wall motion parameters in normal subjects undergoing rest/regadenoson stress using a tagged MRI sequence

**DOI:** 10.1186/1532-429X-18-S1-P91

**Published:** 2016-01-27

**Authors:** Joseph S Soltys, Ibrahim Saeed, James Case, Timothy M Bateman

**Affiliations:** 1 Cardiovascular Imaging Technologies, Kansas City, MO USA; 2 ASPIRE Foundation, Kansas City, MO USA; 3 Saint-Luke's Mid-America Heart and Vascular institute, Kansas City, MO USA

## Background

Changes in global and regional wall motion between rest and stress are important prognostic markers, uncommonly observed with vasodilator stress radionuclide myocardial perfusion imaging (MPI). Cardiac MRI (CMR) with tagging has the potential to more precisely quantify normal and abnormal ventricular wall motion responses. We herein report CMR findings in 5 very low-likelihood patients for CAD undergoing rest/regadenoson stress with tagging.

## Methods

Five females (54 ± 10 yrs) were consented to participate in this Saint Luke's IRB approved research protocol. All had a prior normal rest/regadenoson stress Rubidium-82 MPI (normal perfusion, quantitative flow (ImagenQ, CVIT, Kansas City, MO), rest and peak stress wall motion, and coronary calcium score of 0), within 60 days of participation. CMR images were acquired on a 1.5 Tesla Signa HDxt (General Electric) and 8 channel coil during a single visit with rest imaging preceding stress. Late Gadolinium Enhancement (LGE) images were acquired last to determine if infarcted tissue contributed to any local changes in ventricular motion.

An FRGE-based SPAMM sequence was acquired along the short axis of the left ventricle (LV) at basal, mid, and apical locations at both rest and stress in place of MPI. Typical imaging parameters included a 256 × 160 matrix, 20 phases, a flip angle of 5° and views per segment adjusted to heartrate. Stress imaging began at the peak heart rate response, approximately 30 seconds after manual administration of regadenoson (0.4 mg) and saline flush. An average increase of 34 ± 9 BPM was observed.

Tag line intersections were identified and recorded at both end-diastole (ED) and end systole (ES) with ImageJ (NIH) software. The angle of twist was defined as the angular change of a ray projected between the identified tag intersection and the center of the LV cavity from ED to ES, and calculated for each slice location at both rest and stress. A two-sided students t-test with paired data was used to determine significance (a = 0.05).

## Results

**Figure 1:**
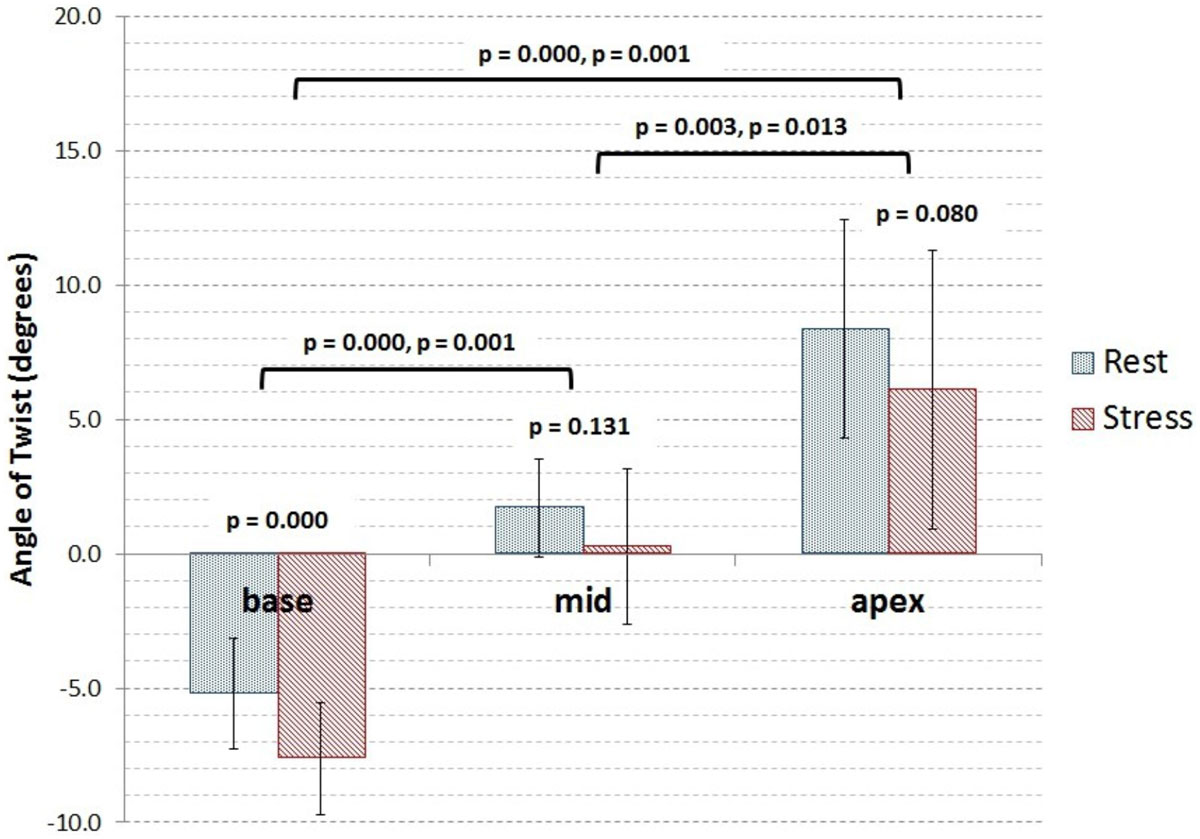
**Average change in ventricular twist between ES and ED per slice location**. Errors bars are ± standard deviation. Negative values indicate clockwise rotation (CW), positive values indicate count-clockwise rotation (CCW). P-Values with bars are for between slice student's t-test comparisons (rest and stress respectively), P-values without are for within slice comparison between rest and stress with alpha = 0.05.

Clockwise (CW) or Counter clock-wise (CCW) average twist at rest and stress for each slice were as follows: basal 5.2 ± 2.1° v 7.6 ± 2.1° (CW, *p* = 0.000); mid-ventricular 1.7 ± 1.8° v 0.3 ± 2.9° (CCW, *p*=0.13); and apical 8.4 ± 4.0° v 6.1 ± 5.2 ° (CCW, *p*=0.08). Rest images were in agreement with previously reported values (4). Significant differences were observed between all combination of slice locations at both rest and stress, within the slice location at the base, and a trend towards the apex (Figure 1).

## Conclusions

This study establishes a baseline for regadenoson-induced changes in ventricular twist in low-likelihood for CAD subjects suggesting the usefulness of SPAMM derived indices in evaluating ischemic populations.

